# Use of health services according to income before and after elimination of copayment in Germany and restriction of universal health coverage in Spain

**DOI:** 10.1186/s12939-018-0725-0

**Published:** 2018-01-27

**Authors:** Lourdes Lostao, Siegfried Geyer, Romana Albaladejo, Almudena Moreno-Lostao, Elena Ronda, Enrique Regidor

**Affiliations:** 1 Department of Medical Sociology, Universidad Pública de Navarra, Campus de Arrosadía s/n, 31006 Pamplona, Navarra Spain; 2 Medical Sociology Unit, Hannover Medical School, Hannover, Germany; 3 Department of Preventive Medicine and Public Health, Universidad Complutense de Madrid, Madrid, Spain; 4 Universidad de Navarra, Pamplona, Spain; 5 Department of Preventive Medicine and Public Health, Universidad de Alicante, Alicante, Spain; 6 CIBER Epidemiología y Salud Pública (CIBERESP), Madrid, Spain; 7 Instituto de Investigación Sanitaria del Hospital Clínico San Carlos (IdISSC), Madrid, Spain

**Keywords:** Use of health services, Inequalities, Income position, Germany, Spain, Great recession, Copayment

## Abstract

**Background:**

In Germany copayment for medical consultation was eliminated in 2013, and in Spain universal health coverage was partly restricted in 2012. This study shows the relationship between income and the use of health services before and after these measures in each country.

**Methods:**

Data were taken from the 2009 and 2014 Socio-Economic Panel conducted in Germany, and from the 2009 and 2014 European Health Surveys in Spain. The health services investigated were physician consultations and hospital admissions, and the measure of socioeconomic position used was household income. The magnitude of the relationship between socioeconomic position and the use of each health service in people from 16 to 74 years old was estimated by calculating the percentage ratio using binary regression.

**Results:**

In Germany, after adjusting for age, sex, and need for care, in the model comparing the two lower income categories to the two higher categories, the percentage ratio for physician consultation was 0.97 (95% CI 0.96–0.99) in 2009 and 0.98 (95% CI 0.97–0.99) in 2014, and the percentage ratio for hospitalization was 1.01 (95% CI 0.93–1.10) in 2009 and 1.16 (95% CI 1.08–1.25) in 2014. In Spain, after adjusting for age, sex, and self-rated health, the percentage ratio for physician consultation was 0.99 (95% CI 0.94–1.05) in 2009 and 1.08 (95% CI 1.03–1.14) in 2014, and the percentage ratio for hospitalization was 1.04 (95% CI 0.92–1.18) in 2009 and 0.99 (95% CI 0.87–1.14) in 2014.

**Conclusion:**

The results suggest that elimination of the copayment in Germany did not change the frequency of physician consultations, whereas after the restriction of universal health coverage in Spain, subjects with lower incomes had a higher frequency of physician consultations.

## Background

In countries with universal coverage of health care, it has been observed that the probability of consulting the general practitioner and of hospitalization either does not vary across income or socio-economic groups or is somewhat more frequent in subjects belonging to lower socioeconomic groups [1–7].

Nevertheless, the economic crisis of 2008 may have had an impact on one of the basic principles underlying the Welfare State, which is equality in the use of health services for the same level of need. The lack of empirical evidence makes it difficult to know whether the crisis has changed the principle of equity in the use of health services [8]. The variety of responses to the economic crisis in Europe offers the possibility of resolving this uncertainty, by investigating what occurred before and during the crisis in Spain and Germany, two countries that implemented different political measures.

Whereas in Germany the annual growth in per capita health expenditure (Purchasing Power Parity) in the public sector between 2009 and 2014 was 4.7% (3229.6 $ in 2009 and 3989.6 $ in 2014), in Spain the growth in the same period was negative: − 1.7% (2301.0 $ in 2009 and 2102.0 $ in 2014) [9]. Furthermore, on 1 January 2013, Germany eliminated the health copayment that had been in effect since 2004 and which obligated patients to pay a fixed amount of 10 euros for each quarter in which they needed health consultation in the public sector [10]. In contrast, in 2012 Spain implemented a measure that restricted the use of public health services, both for Spaniards who were not affiliated with the Social Security and had an annual income of over 100,000 euros, and for immigrants who did not belong to the Social Security system [11–13]. Other restrictive measures were the increase of the copayment in medicines according to the level of income, which in the case of people with annual income equal to or greater than 100,000 euros reached 60% of the retail price.

The objective of this investigation is to show the evolution of the relationship between income and the use of health services in Germany and Spain during the economic crisis, before and after co-payment in Germany was eliminated and restriction measure in the use of public health services in Spain was implemented.

## Methods

### Data sources

The data for Germany were taken from the 2009 and 2014 Socio-Economic Panel (SOEP). The SOEP is a nationwide longitudinal survey project located at the German Institute for Economic Research. The SOEP employs a two-stage stratified sampling design. The regional units of the first sampling stage correspond largely to the electoral districts for the German National Assembly from which households were drawn. A random route sampling point (voting district) was used to select the households. Within each household, all adults aged 16 or over were selected. The first wave was carried out in 1984, and regular follow-ups are conducted to keep up with recent developments. To compensate for panel attrition, new subjects are sampled each year in order to obtain a sufficiently large number of cases and to avoid biases in the composition of respondents. The Spanish data were taken from the 2009 and 2014 European Health Surveys conducted in Spain by the National Statistics Institute. The sampling framework was made up of the Spanish non-institutionalized population aged 16 or over. This survey also had a two-stage sample design. The first-stage units were the census sections, and the second-stage units were the households in each selected section. Households were selected by simple random sampling, and one adult aged 16 or over was selected within each household. Information was collected by face-to-face interviews in both the German and Spanish surveys. In the present study, only subjects under age 75 were selected given that the probability of being institutionalized increases after that age.

### Study variables

The health services investigated in each country were physician consultation and hospital admission. In the SOEP survey, respondents were asked if they had consulted a physician in the last 3 months, and those who answered in the affirmative were asked about the number of consultations. A person was considered to have consulted a physician if they had made any consultation in those 3 months. In the European Health Surveys in Spain, respondents were interviewed about the frequency of their physician visits and had to choose one of the following four alternatives: less than 4 weeks ago, between 4 weeks and a year, more than a year ago, and never. People were considered to have consulted a physician if this had occurred in the last 4 weeks. In both the German and Spanish surveys, respondents were asked if they had been hospitalized overnight at any time during the previous year. Those replying yes were considered to have had a hospital admission.

The measure of income level used was household income. The categories included in this variable for each year are shown in the Table 1. The SOEP database contains several measures of income based on the information on household income obtained from respondents. In this study we used household income weighted by number of household members, in accordance with the recommendations of the Organization for Economic Cooperation and Development. For the statistical analysis, subjects were grouped into four categories using the quartile distribution in the first year, and taking these same cut-off points for the second year. In the European Health Surveys in Spain, household income was not obtained with an open-ended question; rather, respondents had to select an income category from among several intervals shown on the questionnaire. For the statistical analysis, subjects were grouped into four categories. In the Spanish survey, about one-fifth of subjects did not answer the question on income (18% in 2009 and 20% in 2014).

**Table 1:** Categories of household income

Germany	Spain
Income collected by open-ended question in each year and weighted by number of household members	Income collected by closed question with various intervals (different intervals were used in 2009 and 2014)
2009	2009
Greater than or equal to 4000 euros (high)	Greater than or equal to 2400 euros (high)
3999 to 2750 euros (medium-high)	2399 to 1700 euros (medium-high)
2749 to 1851 euros (medium-low)	1699 to 1150 euros (medium-low)
Less than or equal to 1850 euros (low)	Less than or equal to 1149 euros (low)
2014	2014
Greater than or equal to 4000 euros (high)	Greater than or equal to 2040 euros (high)
3999 to 2800 euros (medium-high)	2039 to 1400 euros (medium-high)
2799 to 1960 euros (medium-low)	1399 to 970 euros (medium-low)
Less than or equal to 1959 euros (low)	Less than or equal to 969 euros (low)

Sex and age were used in the analyses as confounding variables, and self-rated health was used as the measure of the need for health care. Age was included as five-year age groups. As in a previous study about income and access to medical care [7], self-rated health has been used as predictor de need for care because this variable have been found to correlate closely with a whole range of other health and healthcare need indicators [14, 15]. In the German survey, self-perceived health was measured by the following question: How would you describe your current health? Respondents had to choose one of the following five alternatives: very good, good, satisfactory, poor or bad. In the Spanish health survey, self-perceived health was measured by the following question: “Over the last 12 months would you say your health has on the whole been very good, good, fair, poor or very poor”. Respondents also had to choose one of these five alternatives. In the analysis of the Spanish data we also included the place of birth as an adjustment variable and we classified the subjects as natives and immigrants.

### Statistical analysis

For each country we estimated the frequency – as a percentage – of respondents who had consulted a physician as well as the percentage of those who had had any hospital admission according to the measure of socioeconomic position. We then estimated the magnitude of the relationship between income and the use of each health service by calculating the percentage ratio estimated by binary regression, taking subjects included in the highest income category as the reference group. The variables included in the regression models as possible confounders and/or as indicators of the need for care were age, sex, and self-rated health. Since income was collected as an interval in the Spanish surveys, it was not possible to develop a weighted income measure for household members. Nevertheless, the analyses of the Spanish survey data also included household size (number of members) as a possible cofounder when the measure of socioeconomic position was household income. Finally, to show a simple measure of the magnitude of socioeconomic differences in the use of health services in each country, we also estimated a summary measure comparing the two lower income categories combined with respect to the two higher income categories.

## Results

Table 2 shows the distribution of the population according to the frequency of physician consultation and hospitalization by income in Germany and Spain. In Germany, the percentage of persons who had consulted a physician or had been hospitalized decreased between 2009 and 2014, except for those in the lowest income category for physician services, and persons in the highest and lowest income categories for hospitalization, in which the percentage increased. In Spain, the percentage of persons who had consulted a physician increased in all income categories between 2009 and 2014, while the percentage of those who had been hospitalized decreased.

**Table 2:** Sample size and frequency (in percentage) of physician consultations by household income. Germany and Spain, 2009 and 2014

Country and categories of household income	Sample size (n)		Physician consultation^a^ (%)		Hospital admission^b^ (%)	
	2009	2014	2009	2014	2009	2014
Germany						
High	4496	5675	68.9	66.9	8.9	9.2
Medium-high	4472	5433	70.1	67.1	11.8	10.7
Medium-low	4455	4911	72.1	68.3	13.0	11.8
Low	4471	5337	71.4	72.4	12.8	15.8
Spain						
High	3542	3906	27.9	28.2	7.2	5.7
Medium-high	3407	4053	28.8	31.6	7.5	6.7
Medium-low	3715	3588	32.1	35.1	8.6	7.9
Low	4943	3947	36.1	39.4	9.7	7.9

^a^Physician consultation refers to the last 3 months in Germany and to the last 4 weeks in Spain

^b^Hospital admission refers to the last year

The relationship between income and physician consultation is shown in Table 3. In Germany, the percentage ratio adjusted for age and sex showed no significant differences in any of the income categories with respect to the highest category. After adjusting for age, sex, and self-perceived health, only the lowest income category was statistically different from the highest income category: 0.94 [95% confidence interval (95% CI) 0.92–0.97] in 2009 and 0.97 (95% CI 0.95–0.99) in 2014. In Spain, the age- and sex-adjusted percentage ratio was highest and was statistically significant in the two lower income categories. After adjusting for age, sex and self-perceived health, the percentage ratio in the different income categories was not significantly different from the reference income category in 2009, but it was in 2014. The percentage ratio in the two lower income categories in 2014 was 1.08 (95% CI 1.02–1.15) and 1.13 (95% CI 1.06–1.20), respectively. The percentage ratio adjusted for age, sex and self-perceived health that compared the two lower income categories with the two higher categories was 0.97 (95% CI 0.96–0.99) and 0.98 (95% CI 0.97–0.99) in 2009 and 2014, respectively, in Germany, and was 0.99 (95% CI 0.94–1.05) and 1.08 (95% CI 1.03–1.14), respectively, in Spain.

**Table 3:** Physician consultation by household income in Germany and Spain. Percentage ratio (PR) and 95% confidence interval (95% CI)

Country and household income	2009				2014			
	Model 1		Model 2		Model 1		Model 2	
	PR	95% CI	PR	95% CI	PR	95% CI	PR	95% CI
Germany								
High	1.00		1.00		1.00		1.00	
Medium-high	1.01	0.99–1.04	0.98	0.96–1.00	1.00	0.98–1.03	0.99	0.97–1.01
Medium-low	1.02	0.99–1.04	0.98	0.96–1.00	1.01	0.99–1.03	0.98	0.96–1.00
Low	0.98	0.96–1.01	0.94	0.92–0.97	1.02	0.99–1.04	0.97	0.95–0.99
The two low vs. the two high categories	0.99	0.98–1.01	0.97	0.96–0.99	1.01	0.99–1.03	0.98	0.97–0.99
Spain								
High	1.00		1.00		1.00		1.00	
Medium-high	1.03	0.96–1.09	1.01	0.95–1.08	1.10	1.04–1.17	1.04	0.98–1.11
Medium-low	1.08	1.01–1.15	1.00	0.94–1.06	1.19	1.12–1.27	1.08	1.02–1.15
Low	1.12	1.05–1.19	0.97	0.91–1.03	1.32	1.24–1.40	1.13	1.06–1.20
The two low vs. the two high categories	1.07	1.02–1.13	0.99	0.94–1.05	1.20	1.14–1.26	1.08	1.03–1.14

Model 1. Adjusted for age and sex

Model 2. Adjusted for age, sex, and self-perceived health

The relationship between income and hospitalization is shown in Table 4. In Germany, the percentage ratio adjusted for age and sex was significantly higher in all income categories than in the highest income (reference) category. After adjusting for age, sex and self-perceived health, the percentage ratio decreased; it was highest -- and statistically significant -- in the category of medium-high income in 2009 and in the lowest income category in 2014: 1.34 (95% CI 1.20–1.49). In Spain, after adjusting for age, sex and self-perceived health, the percentage ratio in the different categories of income did not show significant differences with respect to the highest income category in either of the two periods. The percentage ratio adjusted for age, sex and self-perceived health that compared the two categories of lower income to the two higher income categories was 1.01 (95% CI 0.93–1.10) and 1.16 (95% CI 1.08–1.25) in 2009 and 2014, respectively, in Germany, and was 1.04 (95% CI 0.92–1.18) and 0.99 (95% CI 0.87–1.14), respectively, in Spain.

**Table 4:** Hospitalization according to household income in Germany and Spain. Percentage ratio (PR) and 95% confidence interval (95% CI)

Country and household income	2009				2014			
	Model 1		Model 2		Model 1		Model 2	
	PR	95% CI	PR	95% CI	PR	95% CI	PR	95% CI
Germany								
High	1.00		1.00		1.00		1.00	
Medium-high	1.26	1.12–1.41	1.18	1.05–1.32	1.16	1.04–1.29	1.09	0.98–1.22
Medium-low	1.24	1.10–1.40	1.12	1.00–1.26	1.22	1.10–1.37	1.11	1.00–1.24
Low	1.21	1.07–1.38	1.06	0.93–1.20	1.55	1.39–1.72	1.34	1.20–1.49
The low vs. the high categories	1.10	1.01–1.20	1.01	0.93–1.10	1.27	1.18–1.37	1.16	1.08–1.25
Spain								
High	1.00		1.00		1.00		1.00	
Medium-high	1.03	0.88–1.21	1.01	0.87–1.18	1.14	0.97–1.34	1.00	0.85–1.17
Medium-low	1.15	0.99–1.33	1.02	0.88–1.19	1.31	1.12–1.55	1.05	0.89–1.23
Low	1.22	1.05–1.41	1.06	0.91–1.23	1.33	1.13–1.57	0.94	0.80–1.11
The low vs. the high categories	1.13	1.00–1.28	1.04	0.92–1.18	1.25	1.09–1.44	0.99	0.87–1.14

Model 1. Adjusted for age and sex

Model 2. Adjusted for age, sex, and self-perceived health

Table 5 shows the relationship of income to physician consultation and hospitalization in Spain, after adjusting for age, sex, self-perceived health and place of birth. The results are similar to those seen in the preceding tables. The percentage ratio comparing the two lower income categories to the two higher income categories was 1.00 (95% CI 0.95–1.05) and 1.09 (95% CI 1.03–1.14) in 2009 and 2014, respectively, for physician consultation, and 1.04 (95% CI 0.91–1.18) and 0.98 (95% CI 0.85–1.12), in 2009 and 2014, respectively, for hospitalization.

**Table 5:** Physician consultation and hospitalization by houshehold income In Spain. Percentage ratio (PR) and 95% confidence interval (95% CI)^a^

Household income	Physician consultation				Hospitalization			
	2009		2014		2009		2014	
	PR	95% CI	PR	95% CI	PR	95% CI	PR	95% CI
High	1.00		1.00		1.00		1.00	
Medium-high	1.01	0.95–1.08	1.05	0.98–1.11	1.01	0.87–1.18	0.99	0.84–1.16
Medium-low	1.00	0.94–1.07	1.09	1.03–1.16	1.05	0.90–1.22	1.03	0.88–1.21
Low	0.98	0.92–1.04	1.14	1.07–1.21	1.05	0.90–1.22	0.91	0.77–1.08
The two low vs. the two high categories	1.00	0.95–1.05	1.09	1.03–1.14	1.04	0.91–1.18	0.98	0.85–1.12

^a^Adjusted for age, sex, self-perceived health and place of birth

## Discussion

### Main findings

Between 2009 and 2014, the frequency of physician consultation and of hospitalization decreased in Germany, except in persons belonging to the lowest income category. In Spain, the frequency of physician consultation increased between the first and second period, whereas the frequency of hospitalization decreased. In Germany, after adjusting for age, sex and need for care, subjects in the lowest income category showed the lowest frequency of physician consultation in both periods. In contrast, no significant differences by income were seen in hospitalization in 2009, whereas subjects belonging to the lowest income category showed the highest frequency of hospitalization in 2014. In Spain, after adjusting for age, sex and need for care, no significant differences by income were observed in the frequency of physician consultation in 2009 or in the frequency of hospitalization in either of the periods, while subjects in the lower income categories showed the highest frequency of physician consultation in 2014.

### Comparison with other studies and possible explanations

The reduced frequency of physician consultations in Germany stands in contrast to the increase observed in the two previous decades. The exception was the group with lowest income, which showed a slight increase in the frequency of consultations between 2009 and 2014. The elimination of copayment in 2013 probably contributed to the increased frequency of consultations in this population group. In any case, elimination of copayment did not modify the economic pattern of physician consultation, since in both periods the frequency of physician consultation was lower in the lower income groups. The economic pattern in physician consultation observed in the present study has been found in international comparative studies in various countries [7, 16, 17]. However, some previous investigations in the German population have not found a clear relationship between income and frequency of physician consultations [18] or a greater number of physician visits in persons with lower incomes [19].

Although there is a medical copayment in Germany for hospitalization (10 euros per day for admission up to a maximum of 28 days a year) [20], a previous study found a greater number of hospitalizations in subjects with lower income [20]. In the present study, after adjusting for age, sex and need for medical care, this finding is due to an important increase in the frequency of hospitalization in this population group in 2014 with respect to 2009. The reasons for this finding are unknown. No socioeconomic differences have been found in hospitalization of children and adolescents in Germany, except for the most severe health problems, which showed a longer duration of hospitalization in those in lower socioeconomic position [21]. The slight increase observed in the frequency of physician consultations in persons with lower incomes may be due to patients with more severe health problems, with a consequent increase in the frequency of hospitalization in this population group. In any case, it is possible that one year is not a sufficient period of time to identify changes in the behaviour of patients due to the very recent (2013) elimination of the co-payment.

In Spain, as observed in previous studies, no economic differences were found in the frequency of hospitalization in the first years of the present century [22, 23]. These investigations also have failed to find a clear relationship between income and the frequency of physician consultations [22, 23]. On the other hand, a surprising finding in our study is the increased frequency of physician consultations in Spain. Not only because this increase contrasts with the reduced frequency observed since the beginning of this century, but also because it occurred despite the reduction in health care expenditures and the fact that access to health care was restricted in part of the immigrant population. The increase in physician consultations was particularly notable in the lower income groups. In fact, after adjusting for all the different variables, no economic differences were observed in the frequency of consultations in 2009, while in 2014 the highest frequency of consultation was seen in the lower income groups. A previous study also found an increased frequency of general practitioner consultations in the lowest social classes between 2006 and 2012 [24]. The increased frequency of physician consultations in our study was similar in both the native and immigrant populations, since adjustment for place of birth did not change the magnitude of the association between income and physician consultation. A previous investigation also showed a similar level of health services use by both immigrants and the native population between 2006 and 2012 [25].

This increased frequency of physician consultation may be due primarily to an increase in visits to specialist physicians, given that the percentage of respondents who consulted a specialist in the last 4 weeks before the interview was 11.8% in 2009 and 14.2% in 2014, whereas the percentage of those who consulted a general practitioner hardly changed (28.5% in 2009 and 29.0% in 2014) [26]. Since the increase in physician consultations was not associated with an increased frequency of hospitalization (which decreased), it may be due to a change in clinical practice on the part of general and/or specialist physicians. It is possible that there has been an increase in the rate of referral of patients from general practitioners to specialist physicians. In Spain the general practitioner is the gatekeeper to the health system, therefore patients cannot see a specialist unless referred by a general practitioner. However, an increased number of “interconsultations” (referral from one specialist to another) should not be ruled out as another explanation. According to information on health care activity in specialist care centres, the number of consultations with specialist physicians per person and year rose from 1.8 in 2010 (the first year with available data) to 2.0 in 2014 [27].

In theory, the elimination of the co-payment in Germany would increase the frequency of the use of health services by citizens with lower incomes, while the restrictive measures in Spain would reduce the frequency of use by those citizens, because immigrants belong mostly to the population group with lower incomes. The other affected group, those who were not affiliated with the Social Security and had an annual income of over 100,000 euros, mostly used private health services and, therefore, their behaviour would not be affected by the restriction. However, the economic pattern of use of health services with these measures was not modified, even in Spain it was favourable to citizens with lower incomes. These findings are relevant for similar contexts, that is, developed countries with an important tradition of public coverage of health care. In these countries, the implementation of measures that affect the accessibility to the health system, in one way or another, may not have the desired impact.

### Strengths and limitations

One strength of this study is that it compares the economic pattern in the use of health services in two countries where the economic crisis has had a different impact and which implemented different health policy measures during the crisis. Furthermore, the same data source was used in each country before and during the economic crisis, so that the variables related to the use of physician services and hospital admissions were the same. In the case of Spain, some respondents did not answer the question on household income. We do not believe that this lack of response has influenced the findings, since the percentage of non-response is similar in the categories of another socioeconomic variable, the level of education (data not shown). In addition the percentage of non-response was similar in both years of the study. On the other hand, the cut-off points for the income categories in the surveys carried out in Spain are different. However, given that the alteration in the distribution of the percentage of subjects assigned to each category in 2014 with respect to 2009 has been of small magnitude, its impact on the results must have been minimal.

It is possible that with the use of other measures of socioeconomic position, such as social class or level of education, the results obtained were not the same. However, the measures implemented in Germany and Spain affect the payment capacity of citizens and, in this sense, the measure that best reflects the economic capacity is the level of income. On the other hand, the cut-off points for the income categories in the surveys carried out in Spain are different. However, given that the alteration in the distribution of the percentage of subjects assigned to each category in 2014 with respect to 2009 has been of small magnitude, its impact on the results must have been minimal.

The increase in consultations with specialist physicians in Spain could be attributed to a greater frequency of visits to private specialists. However, the proportion of consultations with public and private specialists remained similar in the two study periods [26]. Finally, the analyses did not include the respondents’ type of health coverage (public, private or mixed) because the European Health Surveys in Spain in 2009 did not include any question about this subject. Nevertheless, adjustment for the type of health coverage in Germany in 2009 and 2014, and in Spain in 2014 did not modify the results.

## Conclusion

In summary, elimination of the copayment for physician visits in Germany did not alter the frequency of consultation according to income, whereas in Spain, after the reduction in health expenditure in the public sector and exclusion of part of the population from health coverage, the frequency of physician consultation was found to increase, especially in the lower income population.

## Abbreviation

SOEP: Socio-Economic Panel
